# A novel method for establishing a mouse model of hepatic echinococcosis: Ultrasound-guided percutaneous liver puncture modeling

**DOI:** 10.1371/journal.pntd.0014154

**Published:** 2026-03-25

**Authors:** Jingyu Wang, Jian Dong, Huijiao Jiang, Huan Zhao, Shengwen Lv, Zhen Wang, Xinhui Cao, Chenghao Liu, Xueling Chen, Xiangwei Wu

**Affiliations:** 1 Department of Hepatobiliary Surgery, the First Affiliated Hospital, Shihezi University, Shihezi, Xinjiang, China; 2 Shihezi University School of Medicine, Shihezi, Xinjiang, China; 3 NHC Key Laboratory of Prevention and Treatment of Central Asia High Incidence Diseases, the First Affiliated Hospital/Shihezi University School of Medicine, Shihezi, Xinjiang, China; National Institute of Allergy and Infectious Diseases Division of Intramural Research, UNITED STATES OF AMERICA

## Abstract

**Introduction:**

Research on *hepatic echinococcosis* relies on stable, reproducible animal models. Traditional open-laparotomy modeling presents issues such as significant trauma and poor consistency.

**Objective:**

To compare the efficacy and safety of ultrasound-guided percutaneous liver puncture inoculation versus traditional open-laparotomy in establishing a *hepatic echinococcosis* model in C57BL/6 mice.

**Methods:**

Alveolar echinococcus (AE) cystic larvae or cystic echinococcus (CE) scolices were implanted into mouse livers using ultrasound-guided percutaneous liver puncture inoculation and traditional open surgery, respectively. Postoperative dynamic ultrasound monitoring, histopathology, and immunofluorescence staining were employed for systematic evaluation.

**Results:**

The survival rate in the ultrasound-guided group reached 100% (30/30), significantly higher than the 80% (24/30) in the surgical group, representing a difference of 20 percentage points (95% CI: 5.7% to 34.3%; p = 0.039). The complication rate was only 6.7% (2/30) in the ultrasound-guided group, compared to 20% (6/30) in the surgical group, with a difference of -13.3 percentage points (95% CI: -30.2% to 3.6%; p = 0.133). Ultrasound-guided modeling achieved a 100% success rate, with more uniform lesion growth and accelerated progression. Histopathology revealed more typical fibrosis and immune microenvironment characteristics.

**Conclusion:**

Ultrasound-guided percutaneous liver biopsy offers advantages including short procedure duration, minimal trauma, and high model consistency. It significantly enhances modeling efficiency while reducing surgical interference, providing a more reliable animal model platform for *hepatic echinococcosis* research.

## Author summary

In this study, we developed a safer and more efficient method utilizing ultrasound-guided percutaneous puncture to establish a *hepatic echinococcosis* model in C57BL/6 mouse livers. Compared to traditional open surgery, this approach requires shorter operative time and causes less trauma, significantly improving mouse survival rates while reducing the risk of complications. Parasite lesions implanted via this technique exhibit more uniform growth and more typical pathological features, better mimicking the fibrosis and immune microenvironment characteristics of human *hepatic echinococcosis.* This modeling strategy provides a stable and reliable animal experimental platform for investigating the pathogenesis of *hepatic echinococcosis* and developing therapeutic strategies, thereby advancing research on this infectious disease.

## Introduction

*Echinococcosis* is a zoonotic disease caused by the parasitism of echinococcal tapeworm larvae, which mainly consists of two types: Alveolar Echinococcus (AE) and Cystic Echinococcosis (CE) [1–3]. Humans become infected by ingesting food or water contaminated with echinococcal eggs, leading to hepatomegaly, abdominal pain, and even multi-organ dysfunction [4–6]. The clinical management of this disease presents several challenges, including low cure rates (only 30% of cases can be treated with surgery, and more than 50% of these cases experience recurrence), limited therapeutic options (the only available drug, albendazole, is associated with significant resistance and hepatotoxicity), and a lack of treatment protocols for patients in the advanced stages of the disease [7–9]. Currently, the mechanisms of larval immune evasion, the host microenvironment and drug resistance are still areas of research. Urgent multidisciplinary collaboration is required to overcome bottlenecks in diagnosis, treatment, and prevention [10].

In experimental studies of hydatid disease, C57BL/6 mice are frequently employed as an animal model. The primary modelling methods employed include the oral administration of hydatid eggs, the intraperitoneal injection of scolex segments, the portal vein injection of scolex segments into the liver, and surgical laparotomy with subcapsular injection of scolices into the liver [11–14]. Oral administration of eggs effectively simulates natural infection and requires minimal technical skills. This method involves mixing eggs into feed or gastric gavage, making the process straightforward. However, infection stability depends on factors like egg viability and gastric acid degradation, leading to prolonged incubation and slow lesion development. Although injecting the scolices into the abdomen is convenient and quick, this method may cause inaccurate injection sites, extended modelling time, and failure to replicate the natural disease progression [11,15]. Portal vein injection of head segments directly delivers scolices to the liver and significantly increases encapsulation rates. However, this technique requires advanced technical skills and precise manipulation. Portal vein injection requires specialised microsurgical skills; incorrect execution can cause haemorrhage, thrombosis, or even death in mice [13,14,16]. Furthermore, the invasive procedure may trigger inflammatory responses that obscure the pathological changes specific to *Echinococcosis*. The subcapsular inoculation of the scolices is currently the most widely employed modelling technique. This technique offers several advantages, including high modelling success rates and precise localisation [17,18]. However, the procedure necessitates open abdominal surgery, which carries a number of risks, including exposure to infection, hepatic injury, and prolonged postoperative recovery periods. The inflammatory response induced by surgery has the potential to exert a significant impact on other intra-abdominal organs, thereby affecting the final experimental outcomes [19]. Therefore, future research must focus on optimising modelling techniques to improve the uniformity and standardisation of animal models.

Based on this, the present study proposes the following hypothesis: Compared with traditional open surgery, ultrasound-guided percutaneous liver puncture inoculation can establish a *hepatic echinococcosis* model in C57BL/6 mice with higher success rates, more uniform lesion development, and more typical pathological features, while causing less trauma and fewer complications. This technique also better mimics the fibrosis and immune microenvironment observed in *hepatic echinococcosis*. To validate this hypothesis, we systematically compared differences between the two modeling approaches in terms of mouse survival rates, complications, modeling success rates, lesion growth dynamics, and histopathological features. Our aim is to establish a more efficient, stable, and less-disturbing animal model platform for *hepatic echinococcosis* to facilitate research into its pathogenesis and the development of therapeutic strategies.

## Materials and methods

### Ethics statement

This animal study was approved by the Ethics Review Committee of Shihezi University (License No.: A2024-427). Informed consent from patients was not required for this study.

### Study animals and materials

Pathogen-free female C57BL/6 mice, aged 6–8 weeksand weighing 18–22 g, were purchased from the Laboratory Animal Center of Xinjiang Medical University and were raised in the animal facility of the College of Medi-cine, Shihezi University. Mongolian gerbils (*Meriones meridianus Pallas*) maintainedin in our laboratory were used for maintaining larval AE. Main instruments: microscope (Axio Imager 2) Zeiss Bio, pipette from Eppendorf, Germany, CO_2_ incubator (Heracell 240i) Thermo Fisher, USA, Doppler ultrasound visualiser (Resona A20T), high-frequency ultrasound probe (33 MHz, the lateral resolution is 100 micrometers, Resona A20T, Mindray, China), Main Reagents: Penicillin and Streptomycin (Solarbio, China), Anti F4/80 Rabbit pAb Q61549, Anti TGF-β Rabbit pAb P04202, 20 × Citric Acid Antigen Repair Solution G1202 (Servicebio, China)

### Grouping design

SPF-grade female C57BL/6 mice (6–8 weeks old, weighing 18–22 g) were selected and randomly divided into two groups: ultrasound-guided group (n = 30), consisting of 15 mice with the hepatic AE model and 15 mice with the hepatic CE model; and traditional surgery group (n = 30), similarly composed. The sample size of n = 30 per group was determined based on prior power analysis (using G*Power 3.1 software) to detect significant differences in primary outcome measures (e.g., survival rate or lesion growth rate) with an effect size of 0.8, α = 0.05, and power (1-β) of 0.8, while accounting for potential attrition during the extended modeling period.

### Extraction of echinococcal scolex

Extraction of AE scolices: Mongolian gerbils infected for 90 days in the laboratory were euthanized under anesthesia with 3% isoflurane mixed with oxygen. Sterile tissue samples were collected via laparotomy. The tissue was washed with PBS (containing 10,000 U/mL penicillin and 10,000 μg/mL streptomycin), homogenized, and passed through a 100-mesh sieve. The precipitate was collected as high-purity scolices [20].

**Fig 1:**
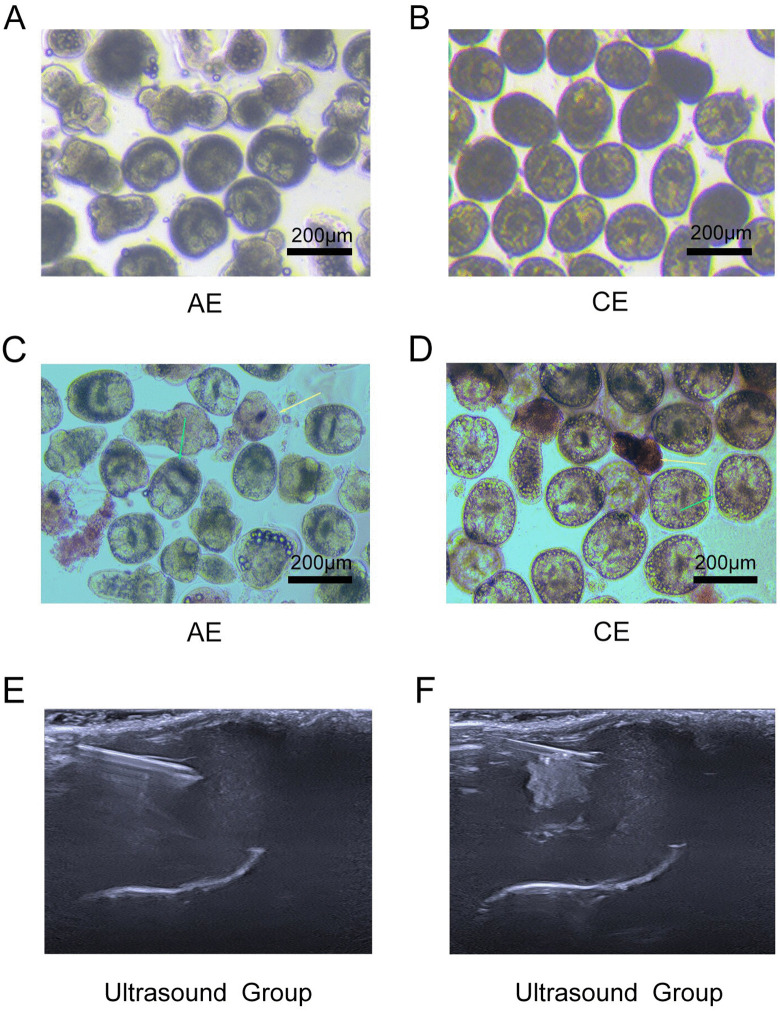
Process flow for extracting the scolex of AE and CE and establishing a mouse model of echinococcosis: A-B show the scolices of AE and CE, respectively, isolated from infected hosts at 40  × magnification. **C-D** The viability of AE and CE was assessed using 0.1% eosin staining, isolated from infected hosts at 40 × magnification.The yellow arrow indicates dead scolices completely stained red. The green arrow indicates unstained scolices with high viability. **E** presents real-time ultrasound imaging during percutaneous hepatic injection guided by ultrasound. **F** depicts immediate post-injection ultrasound imaging following successful scolex suspension injection.

Extraction of CE scolices: Fresh CE mass from sheep liver obtained from slaughterhouses in Xinjiang, belonging to *the sheep strain (G1) of Echinococcus granulosus* [21]*.* After extracting mass fluid and sedimentation, the supernatant was discarded in a laminar flow cabinet, and the sediment layer was collected. The sediment was purified by repeated sedimentation with PBS [22]. All scolices were tested with 0.1% eosin staining to confirm viability exceeding 85% prior to use (Fig 1A,1B,1C, and 1D).

### Ultrasound-guided group modelling method

Firstly, the viable scolices were diluted with PBS solution at concentrations (30,000 AE per ml, 50,000 CE per ml). Mouse were anesthetized using 3% isoflurane mixed with oxygen. After the mouse were anesthetized, the maintenance dose was 2.5%. A skin preparation knife was used to remove 3x4 cm of mouse hair centered on the subxiphoid process, the mouse were placed in a truncated position, the coupling agent was evenly applied, and a high-frequency line-array probe was used to scan the hepatic area, observe the distribution of intrahepatic blood vessels, and determine the path of ultrasound-guided puncture and inoculation.

Using a 1 ml syringe(Needle gauge: 27G, length: 25mm), the needle was inserted obliquely into the liver parenchyma for about 0.5 cm, and the needle could be seen entering into the liver parenchyma under ultrasound (taking care to avoid the intrahepatic blood vessels), and 0.1 ml of echinococcal scolices was injected, and the inoculated scolices could be seen to be clustered into a high echogenic mass inside the liver under ultrasound(Fig 1E and 1F). After removing the needle, apply pressure to the puncture site with a cotton swab for 10 seconds to prevent bleeding. Mouse were rewarmed to 38°C with an electric blanket until they regained consciousness. Ultrasound examinations were conducted at every 30d to monitor lesion growth.

### Surgical group modelling method

Following anaesthesia of the aforementioned mice, a suspension of pre-operative viable scolices was prepared at standard concentration gradients (30,000 AE per ml, 50,000 CE per ml). The surgical field was rigorously disinfected. Expose the left hepatic lobe via a subxiphoid abdominal wall incision using a layered dissection technique. Perform a subepithelial intrahepatic puncture and inject 0.1 milliliters of the suspension. Standard haemostasis procedures were performed post-puncture. Mouse were rewarmed to 38°C with an electric blanket until they regained consciousness. Ultrasound examinations were conducted at every 30d to monitor lesion growth [22].

### Dynamic monitoring and evaluation

Survival rate and complications: Observe the activity, feeding behaviour, and incision status of mice post-modelling. Precisely record time of death. Through gross dissection combined with histopathological examination, differentiate between surgery-related mortality (e.g., haemorrhage, infection), anaesthetic complications, or progression of underlying disease. Lesion Monitoring: Conduct regular ultrasound examinations every 15 days post-modelling to confirm successful modelling and document initial lesion characteristics. Measure lesion dimensions: Determine the long axis (L) and short axis (W) in millimeters. Calculate the approximate volume using the formula V = π/6 × L × W², and compile statistics.

### Pathological validation

According to the experimental design, dissect and collect liver tissue at different stages post-modelling. Immerse in 4% paraformaldehyde (PFA) for 48 hours for fixation. followed by graded ethanol dehydration and paraffin embedding. Perform haematoxylin and eosin (H&E) staining, Lillie’s Modification of Masson’s Trichrome and immunofluorescence to observe cystic structures and inflammatory responses. Assess necrotic debris, cystic structures (small round sacs), calcifications (blue-purple granules), and inflammatory response. This study employed a multiplex immunofluorescence staining method based on tyramide signal amplification (TSA) technology to observe the expression of F4/80 and TGF-β in the lesion areas. Paraffin-embedded sections were embedded in water and subjected to antigen retrieval; frozen sections were fixed with methanol. The brief workflow is as follows: After endogenous peroxidase blocking and serum blocking, sections underwent three rounds of target staining. Each round included primary antibody incubation (overnight at 4°C), corresponding HRP-labeled secondary antibody incubation (50 min at room temperature), and TSA signal amplification (10 min incubation in the dark). Following each round, antibodies were washed away to remove the preceding antibody complex before proceeding to the next staining cycle. Nuclei were counterstained with DAPI, and slides were sealed with an anti-fluorescence quenching mountant after autofluorescence quenching. All images were acquired using a Nikon A1R laser confocal microscope and subjected to semi-quantitative fluorescence intensity analysis via ImageJ software.

### Statistical analysis

Numerical data were expressed as mean ± SEM. Statistical analyses were performed using GraphPad Prism 8 software. Survival rates were compared using the Kaplan-Meier method with the log-rank test; continuous variables were analyzed using the unpaired Student’s t-test or Mann-Whitney U test; categorical variables (e.g., complication rates) were compared with Fisher’s exact test; longitudinal lesion volume data were assessed by one-way/two-way ANOVA with appropriate post-hoc tests; and correlations were evaluated using Spearman’s rank correlation coefficient. Statistical significance was defined as *P < 0.05, **P < 0.01, and ***P < 0.001.

## Results

### Mortality and complication rates related to modelling were lower in the ultrasound group than in the surgical group

The overall survival rate in the ultrasound-guided group (n = 30) was 100% (30/30), significantly higher than the 86.7% (26/30) in the surgical group, representing a difference of 13.3 percentage points (95% CI: 1.2% to 25.4%; p = 0.039). All deaths in the surgical group were associated with severe complications: two cases of sepsis secondary to incisional infection, one case of multiple organ failure due to intestinal obstruction, and one case of intestinal necrosis (blackened bowel due to ischemia) caused by intra-abdominal adhesions. The postoperative complication rate in the ultrasound-guided group was only 6.7% (2 cases of puncture site infection), lower than the 20% rate in the surgical group (6 cases; including 4 incision infections, 1 intestinal obstruction, and 1 abdominal adhesion). This represented a difference of -13.3 percentage points (95% CI: -28.9% to 2.3%; p = 0.133). Furthermore, the ultrasound-guided group had shorter mean operative times (5 ± 1 min vs. 12 ± 3 min) and less intraoperative blood loss (< 0.1 mL vs. 0.5 ± 0.2 mL).

### The ultrasound group had significantly higher modelling efficiency and success rate than the surgical group

**Fig 2:**
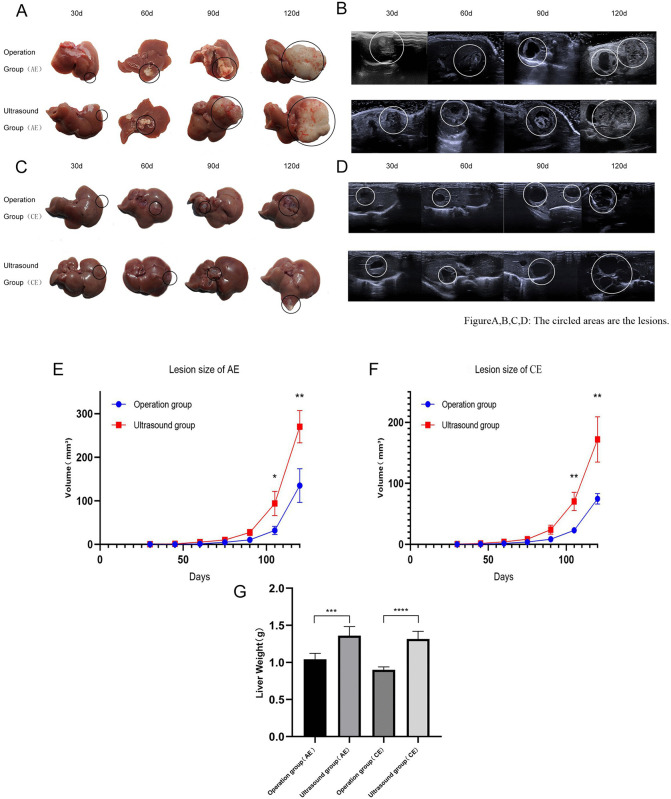
Comparative analysis of hepatic lesion progression in mice infected with AE versus CE at different infection stages. **A** shows representative macroscopic morphology of AE-infected mouse livers at various timepoints (30d, 60d, 90d, 120d). **B** displays corresponding ultrasound images of hepatic lesions. **C-D** show macroscopic morphology and ultrasound images of liver lesions in mice infected with CE at the same timepoints. **E-F** depict line graphs of dynamic changes in approximate parasite lesion volume (V = π/6 × L × W²) over 120 days post-infection for AE and CE, respectively. **G** presents a statistical comparison bar chart of wet weight (mean ± standard deviation) of hepatic lesions in mice infected with AE versus CE at 120 days post-infection. Statistical significance was defined as *P < 0.05, **P < 0.01, and ***P < 0.001.

This study systematically compared lesion growth in *hepatic echinococcosis* models established via ultrasound-guided and surgical methods through dynamic ultrasound monitoring and terminal necropsy. Results demonstrated that the ultrasound-guided approach significantly outperformed traditional surgery in both modeling efficiency and success rate. In the AE model, the ultrasound-guided group achieved 100% (15/15) modeling success by day 75 post-modeling, with uniform lesion growth, while the surgical group showed detectable lesions in only 11 animals at the same time point. Clear lesions were detected in 80% (12/15) of animals by day 90, and the number of successfully modeled cases remained at 12 by the end of the 120-day experiment. Final necropsy revealed 3 cases of peritoneal metastasis in the surgical group versus only 1 in the ultrasound-guided group. Similar trends were observed in the CE model: the ultrasound-guided group achieved 100% (15/15) modeling success by day 75. At the same time point, 10 animals in the surgical group showed detectable lesions, reaching 80% (12/15) success by day 90. By the end of the 120-day experiment, the number of successfully modeled animals remained at 12. Final dissection revealed 2 cases of peritoneal metastasis in the surgical group versus 1 in the ultrasound-guided group. Overall, the ultrasound-guided method achieved a 100% modeling success rate (30/30), significantly higher than the surgical group’s 80% (24/30), representing a difference of 20 percentage points (95% CI: 5.1% to 34.9%; p = 0.0093) (Fig 2A,2B,2C, and 2D).

### The morphological course of disease progression was faster in the ultrasound group than in the surgical group

At 30 days post-infection of mouse livers with the scolices of AE, both the surgical and ultrasound groups exhibited milky-white cystic protrusions centered around the injection site. Continuous observation to 60 day srevealed mass formation in the ultrasound group, while the surgical group exhibited a slightly slower progression. After 90 days, cystic infiltration into surrounding tissues became observable, displaying irregular morphology, and mass were also found in the hepatic hilum. Mass in the ultrasound group were larger than those in the surgical group. By 120 days, ultrasound groups mass continued enlarging beyond hepatic boundaries (Fig 2A and 2E) and exhibited metastasis: one intraperitoneal metastasis in the ultrasound group versus three intraperitoneal metastases in the surgical group.

At 30 days post-infection of mouse livers with the scolices of CE, the surgical group exhibited milky-white mass with slight protrusions, whereas the ultrasound group showed protruding milky-white mass spreading peripherally. By 60 days, this spreading process had further intensified. By day 90, multiple mass with increasing translucency were observable in both groups, with the ultrasound group exhibiting more numerous and larger mass, and mass were also found in the hepatic hilum. By day 120 post-infection, both the number and volume of mass increased. The ultrasound group displayed larger cystic lesions (Fig 2C and 2F). Approximately 0.5 ml of cyst fluid could be aspirated from these lesions. We estimated mass volume by measuring the maximum longitudinal diameter (L) and perpendicular transverse diameter (W) through anatomical dissection at different growth stages, then plotted growth curves to perform quantitative histopathological assessment. At the end of the 120-day experiment, the weight of the liver in the ultrasound group was greater than that in the operation group (Fig 2E,2F, and 2G). These results indicate significantly faster disease progression compared to the surgical group.

### H&E staining and Masson’s trichrome staining observations showed a faster pathological process in the ultrasound group

**Fig 3:**
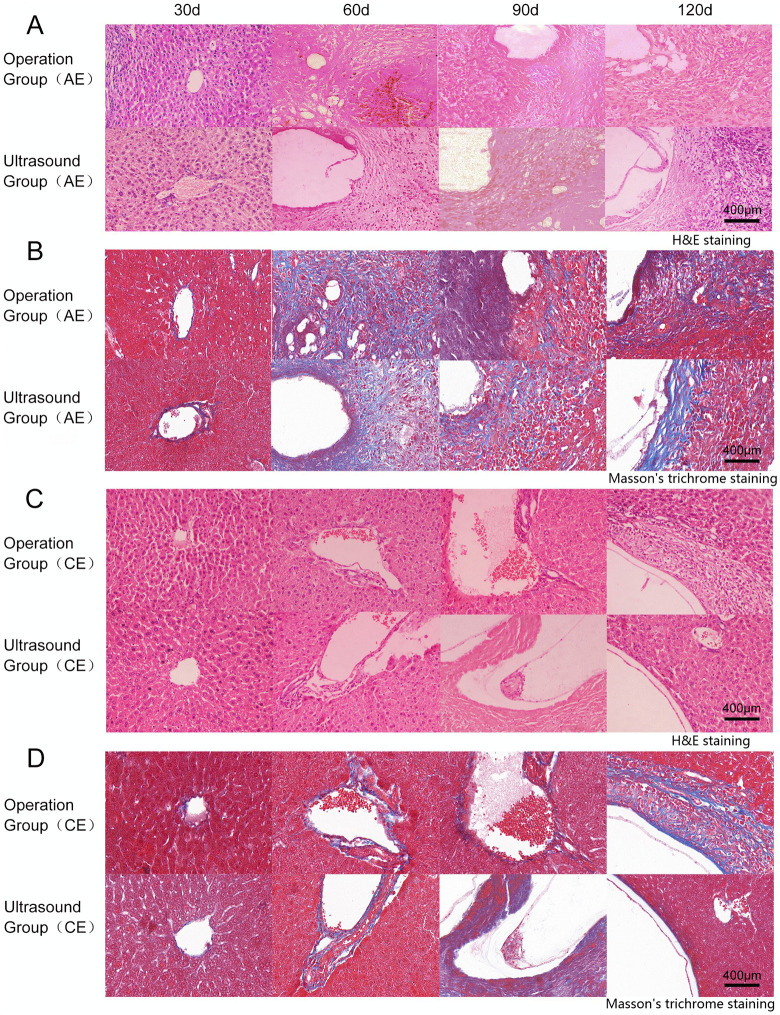
Histopathological analysis of foamy and cystic echinococcal lesions in mouse livers. **A** shows representative H&E staining of AE lesions at different infection stages. **B** presents Masson’s Trichrome staining of the same lesions at 40 × magnification. **C** shows representative H&E staining of CE lesions at different infection stages. **D** presents Masson’s Trichrome staining of the same lesions at 40×.

In the AE groups of hydatid mass, extensive inflammatory cell infiltration, granulation tissue proliferation, and fibrosis were observed at the scolices injection site after 30 days. The mass were relatively small and contained small, round vacuoles surrounded by inflammatory bands, as revealed by microscopic examination. At 60 days, the ultrasound group exhibited mass formation with surrounding tissue infiltration. Meanwhile, the surgical group demonstrated more pronounced inflammatory infiltration, with visible fibrosis and inflammatory cells (neutrophils and lymphocytes). After 90 days, the ultrasound group showed mass infiltration into surrounding tissues and continued mass growth, with both large and small vesicles coexisting, alongside the emergence of fibrous tissue encapsulations, the number of inflammatory cells (neutrophils and lymphocytes) gradually decreases. The surgical group also developed larger vesicles with minor inflammatory infiltration. At 120 days, the ultrasound group exhibited numerous cysts of varying sizes, accompanied by marked tissue destruction and marked tissue destruction with adjacent hepatocyte compensatory hyperplasia. The surgical group also demonstrated mass growth extending into adjacent tissues and fibrosis, though the overall disease progression was slower than in the ultrasound group (Fig 3A and 3B). In summary, during AE development, vesicles and fibrous encapsulations gradually became distinct, exhibiting chronic, infiltrative, tumour-like growth characteristics.

At 30 days in the CE group, both ultrasound and surgical groups exhibited initial cystic remnants at the scolices injection site, with the outer capsule barely discernible. By 60 days, the ultrasound group developed mass tissue with a visible inner capsule wall, surrounded by a thick inflammatory band. The surgical group also formed mass tissue accompanied by perivascular lymphocytic and neutrophils infiltration. At 90 days, the ultrasound group exhibited an intact inner capsule with keratinisation and gradual formation of the outer capsule. The surgical group showed reduced inflammatory bands within the cystic cavity and thickening of the inner capsule compared to the previous period, the lymphocytes and neutrophils in both groups decreased gradually. By 120 days, the ultrasound group demonstrated continued mass enlargement, still enclosed by an intact outer capsule, with the surrounding inflammatory band gradually diminishing until disappearance. The mass exerted marked compression on adjacent hepatic tissues. The surgical group also exhibited mass enlargement and thickening of the outer capsule, though to a lesser extent than the ultrasound group, with a thicker peripheral inflammatory zone (Fig 3C and 3D). During the CE infection process, severe disruption of the hepatic architecture occurred due to inflammatory cell infiltration and progressive fibrosis, accompanied by continuous thickening of the mass wall. No significant infiltration into surrounding tissues was observed.

### Immunofluorescence co-localization of F4/80 and TGF-β expression

**Fig 4:**
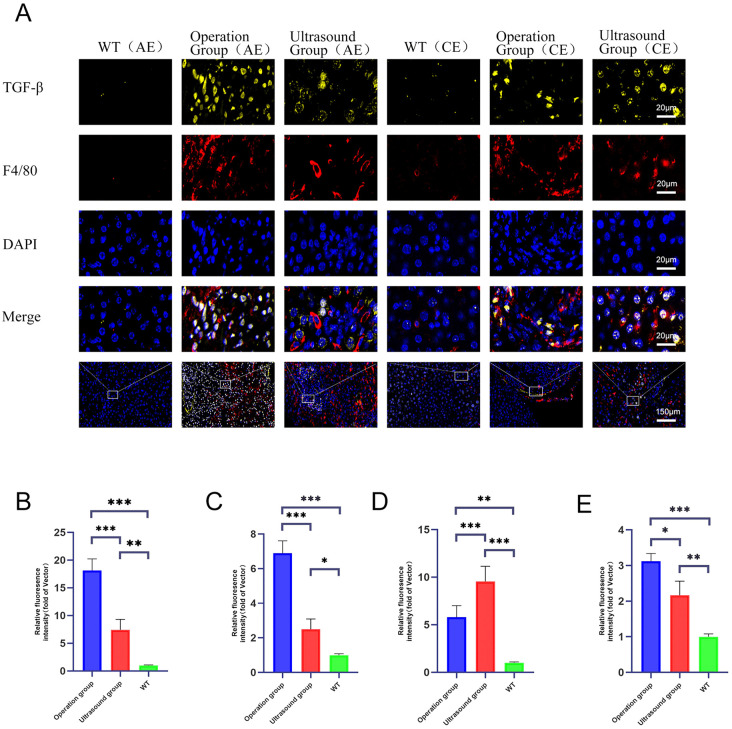
Co-localisation analysis of TGF-β and F4/80 in liver lesions of mice infected with AE and CE. A presents representative immunofluorescence staining of hepatic lesion areas from mice infected with AE and CE at 120 days post-infection (200 × magnification). **B** shows a bar chart of the relative fluorescence intensity ratio of F4-80 to TGF-β in liver tissues from AE, CE, and uninfected control groups (mean ± standard deviation). Fluorescence intensities were normalized and expressed as ratios relative to the baseline (mean of the control group). *P < 0.05, **P < 0.01, and ***P < 0.001 (statistical analysis performed using one-way analysis of variance [ANOVA] followed by post-hoc multiple comparison tests).

At 120 days post-infection, mice from both groups underwent anaesthesia followed by laparotomy. The affected liver tissue was harvested for immunofluorescence colocalization analysis of F4/80 and TGF-β expression. Findings revealed that in the AE group, both F4/80 and TGF-β expression levels were significantly higher in the surgical group than in the ultrasound group, with substantial overlap in the regions of elevated expression (Fig 3A and 3B); this indicates a more robust granulomatous immune response in the surgical group. F4/80, a macrophage marker, exhibited elevated expression, reflecting substantial macrophage infiltration at the infection site. In the CE group, the surgical group exhibited higher F4/80 expression than the ultrasound group, yet lower TGF-β expression (Fig 4A, 4B,4C, and 4D).

## Discussion

This study aims to systematically evaluate the efficacy and safety of ultrasound-guided percutaneous liver biopsy techniques in establishing a hepatic echinococcosis model in mice. By comparing different techniques, we found that ultrasound-guided percutaneous liver puncture significantly improved survival (100%) and lowered complication rates (6.7%) in C57BL/6 mice. In contrast, the conventional surgical approach necessitates a laparotomy incision, resulting in a larger wound that requires healing and may contribute to postoperative stress and infection risk. The ultrasound-guided group achieved a 100% success rate in both hepatic AE and CE models, significantly outperforming the conventional surgical group. These findings highlight the advantages of ultrasound-guided techniques in modelling efficiency and success rates. These results suggest that the choice of modeling techniques is a key experimental variable affecting the quality and stability of the models [23,24].

Moreover, this study used dynamic ultrasound monitoring technology to observe lesion growth in a mouse model of *hepatic echinococcosis*. The observation was real-time, non-invasive, and precise. This method significantly enhanced the efficiency of experimental animal screening and model homogeneity. Compared to traditional evaluation methods reliant on terminal dissection, the ultrasound-guided group enabled longitudinal tracking of lesions at different time points (30d, 60d, 90d and 120d) [25,26], enabling early identification of individuals exhibiting abnormal growth (e.g., mice failing to develop lesions by 60 days), Enhance the homogeneity and screening efficiency of the final model inclusion [23].

Finally, histological analysis revealed faster lesion progression in the ultrasound group compared to the surgical group. The use of ultrasound-guided techniques allows for more precise and concentrated injection of scolices, establishing an initial infection site with a higher concentration of parasites. Conversely, traditional surgical injection may, due to procedural factors, result in more dispersed parasites that are more readily surrounded and confined by localised strong immune responses. Both model groups exhibited a marked increase in lesion size (diameter/volume) growth rate during the later follow-up period. This phenomenon aligns with the natural progression of hydatid disease: in the early stage, parasites establish colonization and reach a temporary equilibrium with the local immune response, resulting in relatively slow lesion expansion; In the middle and late stages, cystic structures (or hydatid mass) gradually mature and stabilize, while parasite proliferation and local microenvironmental remodeling intensify. The model then transitions into an expansion phase, resulting in an accelerated growth trend.

In the context of AE infection, surgical trauma induces a robust inflammatory response [27]. This response is characterised by F4/80  + macrophage infiltration and elevated TGF-ß+ expression [28,29], which attempts to contain the parasite. Conversely, the ultrasound group, due to precise high-dose inoculation, experienced parasite “immune escape” and rapid infiltration [30]. In CE infection, the phenomenon proves more intricate: The surgical group exhibited typical characteristics of the inflammatory phase (high F4/80, low TGF-β), while the ultrasound-guided inoculation group demonstrated an immunosuppressive state characterized by low F4/80 and high TGF-β. Of course, the differences may also stem from injection trauma or local hypoxia, not merely from the dynamics of infection. This differentiated immune microenvironment created more favorable growth conditions for the parasite [31]. This indicates that disease severity does not correlate simply and positively with the intensity of immune markers. Furthermore, this finding highlights that the modelling methodology is a critical experimental variable. It offers significant guidance for future mechanistic research, drug efficacy evaluation, and the development of personalised clinical treatment strategies (e.g., targeting inflammatory or fibrotic patient subtypes).

In practice, this approach is cost-effective and mainly depends on experimental apparatus equipped with ultrasound imaging systems compatible with high-frequency small-animal probes. The practical implementation of this method is greatly enhanced by the widespread commercial availability of high-resolution ultrasound systems that support 33 MHz probes, a technology that has matured since approximately 2015. Technically, researchers with interventional experience may find this method more readily mastered. This study has limitations. The 120-day observation period, while sufficient to document key pathological stages, is shorter than the chronic, multi-year progression of human echinococcosis [32,33]. Furthermore, the exclusive use of female C57BL/6 mice limits insights into potential strain- or sex-dependent variations. Finally, species-specific differences between mice and humans necessitate caution in directly extrapolating the immune and pathological timelines observed here.

In summary, This study compared the modeling efficiency and safety of Echinococcus multilocularis in mouse models using ultrasound-guided techniques versus conventional surgical methods. It demonstrated the significant advantages of ultrasound-guided techniques in enhancing survival rates, reducing complications, and improving the quality of modelling. These findings provide crucial experimental evidence for parasite infection research and lay the groundwork for future clinical applications. Future studies should explore the application of this technique in other animal models and clinical practice to promote advancements in related fields.
